# Predicting sexually transmitted infections among men who have sex with men in Zimbabwe using deep learning and ensemble machine learning models

**DOI:** 10.1371/journal.pdig.0000541

**Published:** 2024-07-03

**Authors:** Owen Mugurungi, Elliot Mbunge, Rutendo Birri-Makota, Innocent Chingombe, Munyaradzi Mapingure, Brian Moyo, Amon Mpofu, John Batani, Benhildah Muchemwa, Chesterfield Samba, Delight Murigo, Musa Sibindi, Enos Moyo, Tafadzwa Dzinamarira, Godfrey Musuka

**Affiliations:** 1 AIDS and TB Programme, Ministry of Health and Child Care, AIDS & TB Programme, Harare, Zimbabwe; 2 Department of Computer Science, University of Eswatini, P Bag 4 Kwaluseni Campus, Swaziland; 3 Department of Medicine, University of Zimbabwe College of Health Sciences, Harare, Zimbabwe; 4 ICAP in Zimbabwe, Harare, Zimbabwe; 5 National AIDS Commission, Harare, Zimbabwe; 6 Faculty of Engineering and Technology, Botho University, Maseru, Lesotho; 7 Gays and Lesbians of Zimbabwe, Harare, Zimbabwe; 8 Sexual Rights Centre, Bulawayo, Zimbabwe; 9 Innovative Public Health and Development, Harare, Zimbabwe; 10 International Initiative for Impact Evaluation (3ie). Harare, Zimbabwe; University of Ulm, GERMANY

## Abstract

There is a substantial increase in sexually transmitted infections (STIs) among men who have sex with men (MSM) globally. Unprotected sexual practices, multiple sex partners, criminalization, stigmatisation, fear of discrimination, substance use, poor access to care, and lack of early STI screening tools are among the contributing factors. Therefore, this study applied multilayer perceptron (MLP), extremely randomized trees (ExtraTrees) and XGBoost machine learning models to predict STIs among MSM using bio-behavioural survey (BBS) data in Zimbabwe. Data were collected from 1538 MSM in Zimbabwe. The dataset was split into training and testing sets using the ratio of 80% and 20%, respectively. The synthetic minority oversampling technique (SMOTE) was applied to address class imbalance. Using a stepwise logistic regression model, the study revealed several predictors of STIs among MSM such as age, cohabitation with sex partners, education status and employment status. The results show that MLP performed better than STI predictive models (XGBoost and ExtraTrees) and achieved accuracy of 87.54%, recall of 97.29%, precision of 89.64%, F1-Score of 93.31% and AUC of 66.78%. XGBoost also achieved an accuracy of 86.51%, recall of 96.51%, precision of 89.25%, F1-Score of 92.74% and AUC of 54.83%. ExtraTrees recorded an accuracy of 85.47%, recall of 95.35%, precision of 89.13%, F1-Score of 92.13% and AUC of 60.21%. These models can be effectively used to identify highly at-risk MSM, for STI surveillance and to further develop STI infection screening tools to improve health outcomes of MSM.

## Author summary

In this study, we investigated the use of machine learning to identify men who have sex with men (MSM) at high risk of sexually transmitted infections (STIs) in Zimbabwe. MSM face a greater risk of STIs due to factors like unprotected sex and limited access to healthcare. We used data from a survey of over 1500 MSM in Zimbabwe to train machine learning models to predict STIs. These models were more accurate than traditional statistical methods at identifying high-risk individuals. Our findings suggest that machine learning could be a valuable tool for improving STI prevention and screening efforts among MSM, particularly in settings with limited resources. This approach could help healthcare providers target interventions to those most in need and ultimately improve the health outcomes of MSM in Zimbabwe.

## 1. Introduction

Among other population groups, men who have sex with men (MSM) are at high risk of sexually transmitted infections and human immunodeficiency virus (HIV) globally [1], accounting for 23% of new adult HIV infections in 2019[2]. Men who have sex with men are more vulnerable to HIV infection than the general population because of their behavioural and biological factors, including Unprotected sexual practices, unprotected anal intercourse[3], multiple sex partners, and substance use [4,5]. These factors, along with criminalization, stigmatisation[6], fear of discrimination [7], poor access to care and lack of early STI screening tools in some countries make MSM more vulnerable and increase the risk of HIV infection [8]. In Zimbabwe, HIV prevalence remains high (11.6%) in the general population [9] and higher (23.4%) among MSM [4]. Several interventions including biomedical and behavioural approaches have been utilised to reduce the risk of HIV and STI transmission in the MSM community. Despite these efforts, STIs remain some of the most common infections among MSM [6]. There is a need to integrate emerging technologies such as machine learning and deep learning, with predictive capacity. By seamlessly incorporating emerging technologies like machine learning and deep learning, we enhance the predictive capabilities of biomedical interventions. This integration can optimise and refine existing approaches by providing more accurate predictions, personalised insights, and timely feedback.

There is significant progress in applying machine learning algorithms to predict HIV incidences [10], diagnosis [6], infections [11], and identifying risk factors associated with HIV infection among MSM. Predicting sexually transmitted infections among MSM is paramount in reducing new infections, improving risk surveillance, and most importantly developing STI pretest screening tools. Such tools can improve STI screening procedures among MSM, especially in resource-constrained settings and in countries where MSM is criminalised and stigmatised. Furthermore, applying machine learning in predicting STIs among MSM can improve STI risk surveillance and also identify those who might require Pre-Exposure Prophylaxis (PrEP), and most importantly identify risk factors associated with STIs among MSM in Zimbabwe. However, there is a dearth of literature on the application of ML models to predict STIs among MSM, especially in developing countries such as Zimbabwe.To address these gaps, this study identified risk factors associated with STIs and predicted STIs among MSM using bio-behavioural data and machine learning models. Unlike statistical models, machine learning models such as logistic regression, artificial neural networks (ANN), support vector machines (SVM), MLP, ExtraTrees, random forest (RF), decision tree, AdaBoost, Bagging and XGBoost have the predictive capacity and the ability to solve complex problems including classification and prediction tasks. Once trained, tested and validated, machine learning models can be utilized to develop intelligent data-driven STI screening applications that can assist hard-to-reach communities such as Men Who Have Sex with Men for early screening of STI with high precision and accuracy and make recommendations for further screening and diagnosis.

## 2. Related work

Machine learning (ML) and deep learning (DL) techniques have made significant progress in improving healthcare services. For instance, machine learning and deep learning models, including recurrent neural network (RNN), neural network convolutional (CNN), random forest, K-nearest neighbours (KNN), logistic regression, decision trees, support vector machine, K-means, and multilayer perceptron have been implemented to solve classification and clustering problems in the public health domain. Existing literature shows that ML and DL models have been mostly used in HIV modelling, predicting HIV incidences [2], HIV infection, and further among MSM, and in some instances developing HIV screening tools [12]. For instance, a study conducted by Makota and Musenge [13] applied catalytic and Farrington models to estimate HIV incidences in Zimbabwe. The findings of their study found that the Farrington models performed better than the catalytic models. Furthermore, Birri Makota and Musenge (2023) [14] also applied the XG Boost algorithm to predict HIV status in Zimbabwe using Demographic Health Survey data from 2005 to 2015. Their study revealed that XGBoost performed better than other models on original data and SMOTE-balanced data. Furthermore, in Australia, Bao et al. [2] applied random forest and gradient-boosting machine learning algorithms to predict HIV and STIs (chlamydia, syphilis, and gonorrhoea) and the models achieved high accuracy. Also, in China, Zhejiang province, He et al. [3] applied support vector machine, decision tree, random forest, and logistic regression to predict HIV infection among MSM.

## 3. Methodology

### 3.1 Data source and ethical considerations

The study used secondary data from the Ministry of Health and Child Care in Zimbabwe. Data were collected as part of an HIV prevalence study conducted by ICAP. From March to July 2019, MSM and transgender women/ gender-queer) (TGW/GQ) individuals were recruited in Harare and Bulawayo, Zimbabwe, for a cross-sectional BBS. MSM and TGW/GQ individuals were eligible for BBS participation if they were born biologically male; engaged in anal or oral sex with a man in the previous 12 months; were aged 18 years or older; resided in Harare or Bulawayo in the previous month; and spoke English, Shona, or Ndebele. The protocol and tools used in this study received Ethical and administrative approvals from the Columbia University Institutional Review Board (# IRB-AAAR8950) and the Medical Research Council of Zimbabwe (#(MRCZ/A/2156). The protocol was reviewed by the US Centres for Disease Control and Prevention (CDC) (# 2018–444). Once eligibility was confirmed, potential participants were provided a copy of the consent form in English, Shona, or Ndebele based on their preference. The survey purpose and procedures, potential risks and benefits of participation, and who to contact to report complaints or concerns were reviewed. Potential participants were informed that participation is confidential and voluntary and that they can withdraw from participation at any time without explaining. Potential participants were provided an opportunity to ask questions. For those who wished to participate, written consent was obtained. The consent form included a separate consent checkbox for each of the two survey elements: 1) completion of the questionnaire, 2) provision of venous blood, storage for future testing, and 3) permission to be contacted by survey staff for follow-up. A copy of the signed consent form was provided to each participant.

## 4. Results

### 4.1 MSM characteristics

A total of 1538 individuals participated in the study. The median age of participants was 25 years (IQR: 21–32). Most participants reported cohabitation without a sex partner (85.18%), 70.61% of participants had completed secondary education, 19.12% had completed tertiary education, and 48.57% reported being employed in the last 6 months. Pentecostalism was the most commonly reported religion (27.96%), followed by Roman Catholicism (19.70%) and no religion (18.79%). Bisexual identity was reported by 40.38% of participants, while 59.62% identified as gay/homosexual, as shown in Table 1. Regarding sexual behaviour, 26.79% of participants reported engaging in transactional sex in the past 6 months. The median number of sexual partners in the past 6 months was 1 (IQR: 1–3), and condom use was reported by 31.47% of participants. Regarding HIV status, 76.14% of participants tested negative, while 22.11% tested positive. Overall, these findings suggest that the study population is a diverse group of young MSM with varying levels of education, employment, and religious affiliation.

**Table 1:** Characteristics of MSM.

Characteristic	n (%)
**Age: median (IQR)**	25 (21, 32)
**Cohabitation with Sex Partner**	
No	1,310 (85.18)
Yes	228 (14.82)
**Education Status**	
None/Primary	82 (5.33)
Secondary	1086 (70.61)
Tertiary	294 (19.12)
Vocational	76 (4.94)
**Employed in the last 6 months**	
No	791 (51.43)
Yes	747 (48.57)
**Religion**	
none	289 (18.79)
Traditional	43 (2.8)
Roman Catholic	303 (19.7)
Protestant	249 (16.19)
Pentecostal	430 (27.96)
Apostolic Sect	84 (5.46)
Other	140 (9.1)
**Sexual Identity**	
Bisexual	621 (40.38)
Gay/Homosexual	917 (59.62)
**Transactional Sex in the past 6 months**	
No	1126 (73.21)
Yes	412 (26.79)
**Number of Sexual Partners in the past 6 Months: median (IQR)**	1 (1, 3)
**Condom Use**	
No	984 (63.98)
Yes	484 (31.47)
Missing	70 (4.55)
**HIV Status**	
Negative	1171 (76.14)
Positive	340 (22.11)
Missing	27 (1.76)

**Fig 1:**
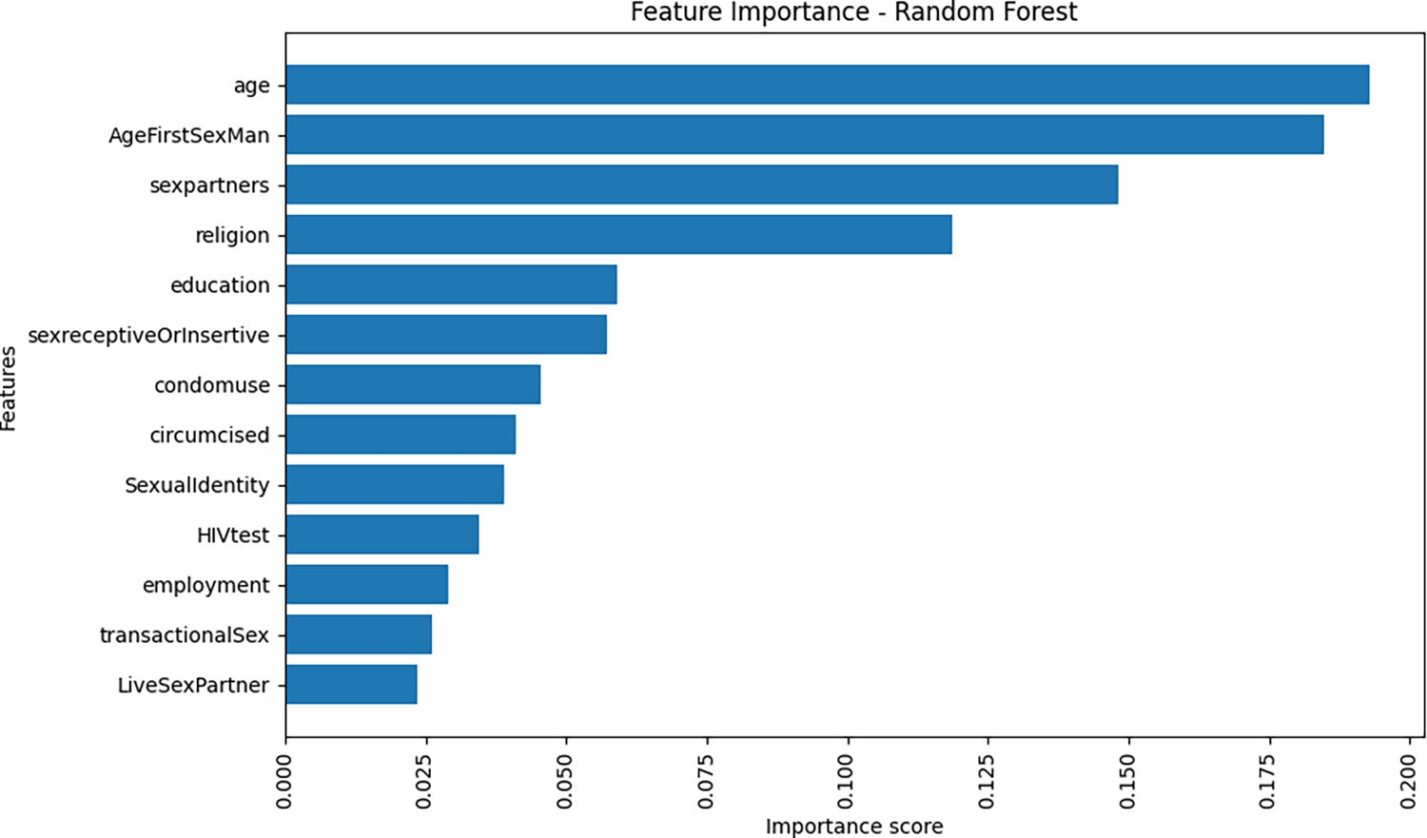
Feature importance score.

In the pursuit of understanding the determinants of STI vulnerability among Men who have Sex with Men (MSM), feature importance analysis, as depicted in Fig 1, identified age, age of first sexual encounter with a man, number of sexual partners, religion, and education as the most important factors. To quantify the strength of influence of these five features, a logistic regression model is employed, and results are shown in Table 2. Individuals aged 26–35 years exhibited 1.35 times higher odds of STIs compared to the reference age group (18–25 years) with a statistically significant p-value of 0.04 (OR = 1.35, p = 0.04). Men initiating sexual activity with a man at ages 36–45 years were 3 times more at risk of STIs compared to men who had their first sexual encounter after the age of 18 years (OR = 3.00, p = 0.03). MSM reporting multiple sexual partners (more than 1) had 1.90 times higher odds of STIs compared to those with a single sexual partner, indicating a strong association (OR = 1.90, p < 0.001). Individuals with a vocational education level exhibited a protective effect against STIs, with an Odds Ratio of 0.45 (p = 0.04), in comparison to those with none/primary education (OR = 0.45, p = 0.04).

**Table 2:** Results from the logistic regression model and STI risk factors among MSM.

Variable	Odds ratio (0R)	p-value	Confidence Interval (CI)
**Current age (years)**			
18–25	Ref		
26–35	**1.35**	**0.04**	**(1.01, 1.81)**
36–45	1.13	0.61	(0.70, 1.82)
46–65	0.99	0.98	(0.47, 2.10)
65+	0.93	0.95	(0.09, 9.57)
**Age at first sex with a man**			
>18	Ref		
18–25	1.15	0.35	(0.85, 1.55)
26–35	0.87	0.57	(0.54, 1.41)
36–45	**3.00**	**0.03**	**(1.14, 7.89)**
46–65	1.43	0.70	(0.23, 8.81)
**Number of sex partners (last 6 months)**			
one	Ref		
multiple	**1.90**	**<0.001**	**(1.46, 2.45)**
**Educational Level**			
None/primary	Ref		
secondary	0.88	0.66	(0.51, 1.53)
tertiary	0.96	0.89	(0.52, 1.77)
vocational	**0.45**	**0.04**	**(0.19, 0.94)**
**Religion**			
Apostolic Sect	Ref		
Traditional	0.55	0.21	(0.22, 1.39)
Christians (Catholics, Protestants & Pentecostal)	0.69	0.17	(0.41, 1.17)
none	0.67	0.17	(0.38, 1.18)
other	0.7115989	0.303	(0.37, 1.36)

### 4.2 Feature engineering and data preprocessing techniques

**Fig 2:**
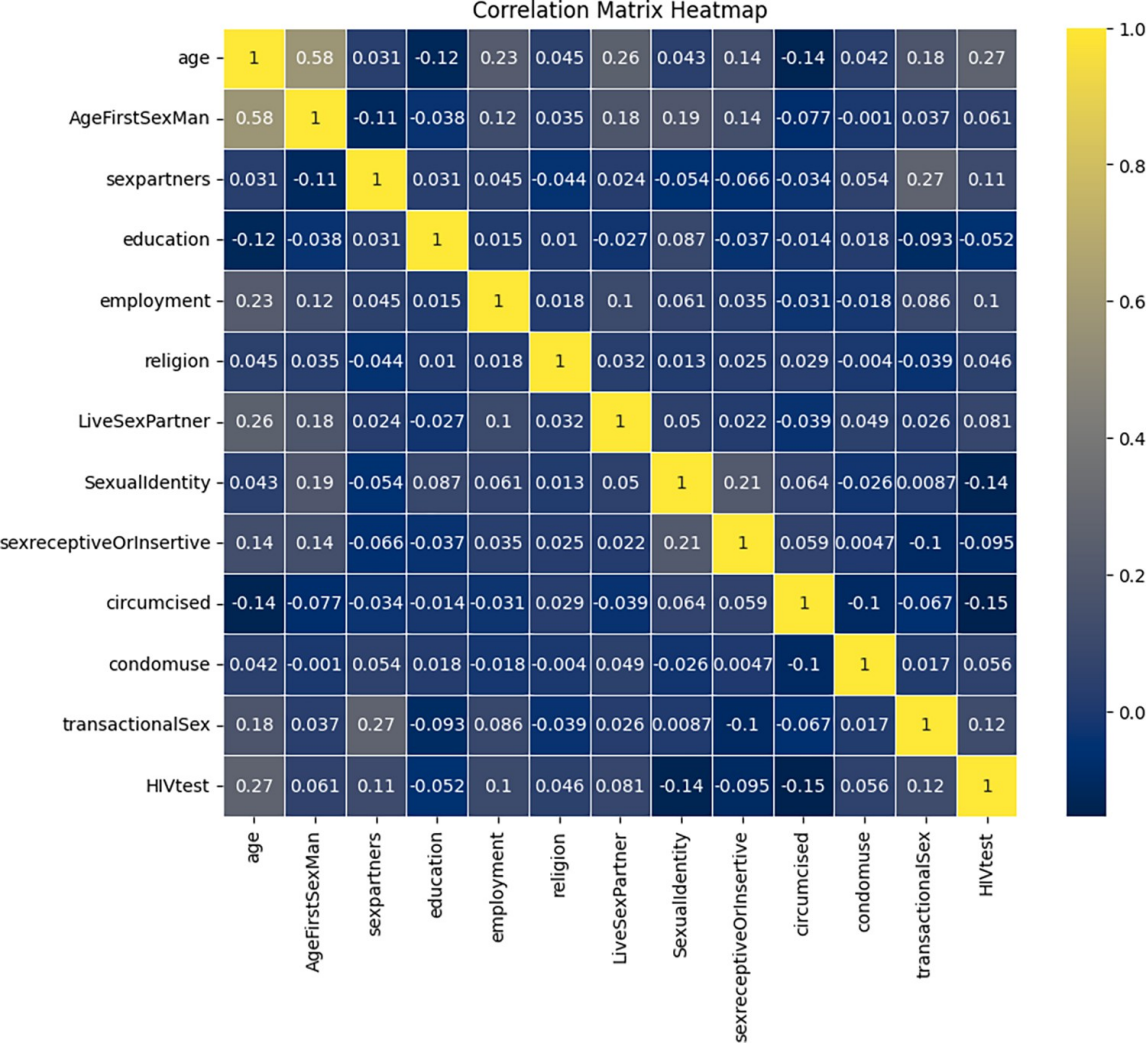
Pearson correlation heatmap.

The study utilised both traditional stepwise logistic regression and machine learning algorithms to identify and assess the significance of various predictors concerning STI. The initial dataset consisted of information from a total population of 1538 MSM. The dataset includes information on various potential predictors and the binary response variable indicating the presence or absence of STI. Out of the pool of 20 potential predictors, a stepwise logistic regression was performed to select the most significant predictors associated with STI. The selected predictors from the stepwise regression included: Age, Cohabitation with Sex Partner, Education Status, Employment Status, Religion, Sexual Identity, Transactional Sex, Number of Sexual Partners in the past 6 Months, Condom Use, and HIV Status. Transactional sex was defined as any exchange of money or goods for sexual acts, whether receiving or giving. All null or missing values were deleted to avoid pollution of samples which may lead to drawing inaccurate inferences when building robust predictive models[15]. After dropping missing values, 1,444 samples were considered for applying STI predictive models. Moreso, features were encoded using one-hot encoding technique to convert categorical data to numerical values. One-hot encoding uses binary vectors to convert variables of categorical features into numerical values [16]. Each category or label in a categorical variable is converted into a binary vector where all elements are zero except for the index corresponding to the category, which is set to 1[17]. One-hot encoding is a simple and widely used encoding method despite creating a high-dimensional feature matrix [17]. Furthermore, the study used Pearson correlation to determine the correlation between the selected explanatory variables and a heat map was drawn as shown in Fig 2.

The study further applied random forest to determine the feature importance score, as shown in Fig 2. The feature importance scores assist in ranking and identifying important features that have the most influence on the predictions of STI infection among MSM. Fig 1 shows that age, age of having first sex with a man, sex partners, religion, and education are among the topmost important features with high-importance feature scores. To quantify the impact of the features with high-importance feature scores, a logistic regression model was employed, aiming to determine the strength of influence these five features exert on the likelihood of STI acquisition among MSM.

**Fig 3:**
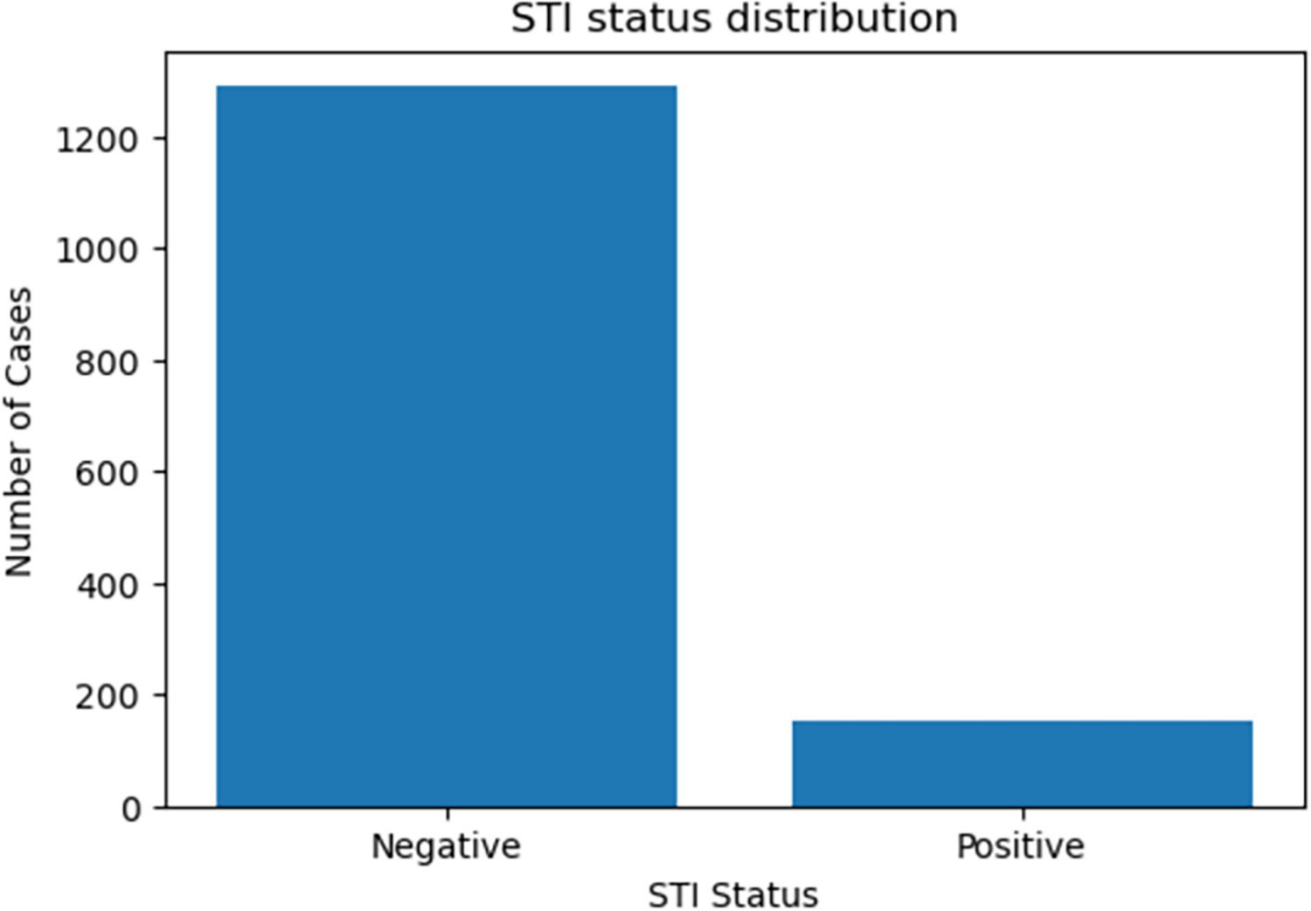
STI status distribution.

From 1,444 samples considered for developing STI predictive models, 1290 tested negative for STI and 154 tested positive. This shows that the target class had class imbalance, as shown in Fig 3. Therefore, the synthetic minority over-sampling technique (SMOTE) technique was applied to address the class imbalance problem. SMOTE creates synthetic samples of the minority class[18]. It uses the K-Nearest Neighbour (KNN) algorithm to identify the nearest data point and creates synthetic data by interpolating between the selected point and its nearest neighbour [19]. This process continues until the minority class is balanced with the majority class in the dataset.

**Fig 4:**
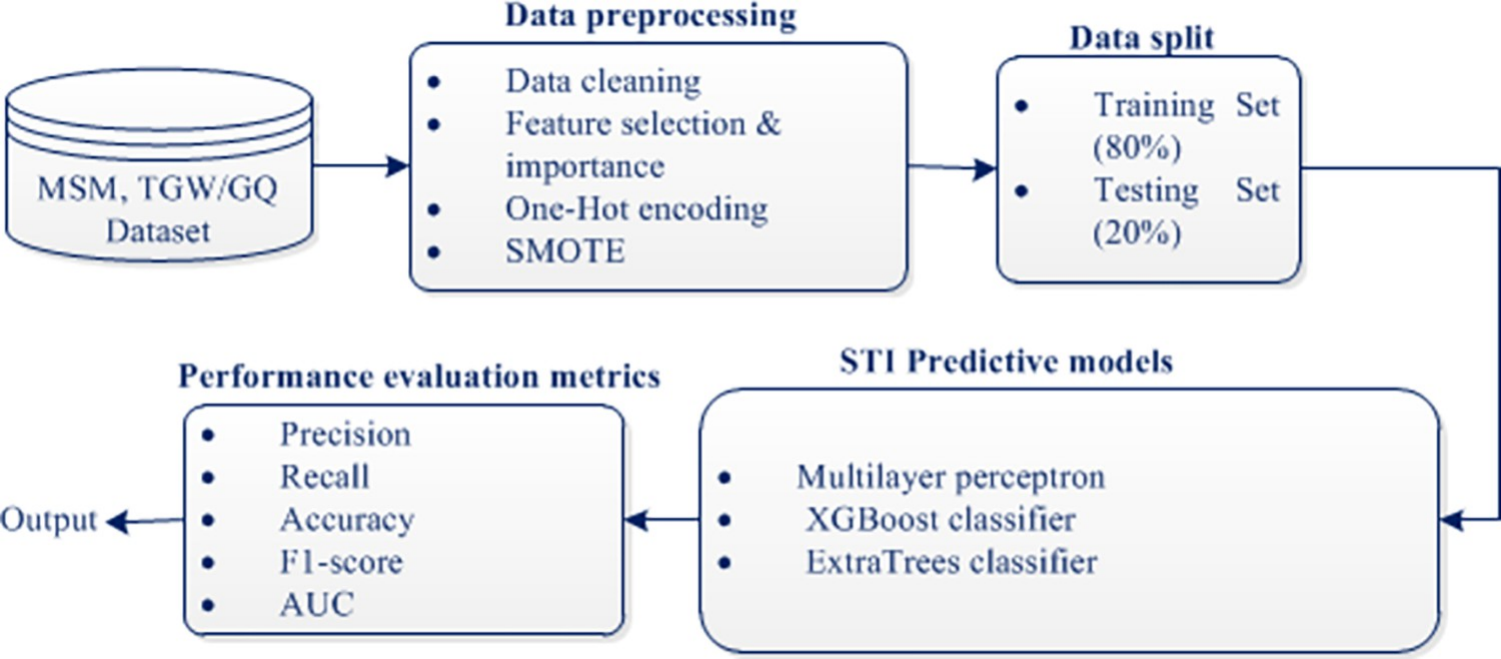
STI predictive models flow diagram.

After handling the class imbalance problem, the dataset was split into the training set and testing set using the split ratio of 80% and 20%, respectively. All the steps followed are shown in Fig 4. The study further applied multilayer perceptron (MLP), ExtraTrees, and XGBoost machine learning models to predict STIs among MSM. These STI predictive models are explained in the subsequent sections.

### Multilayer perceptron (MLP)

**Fig 5:**
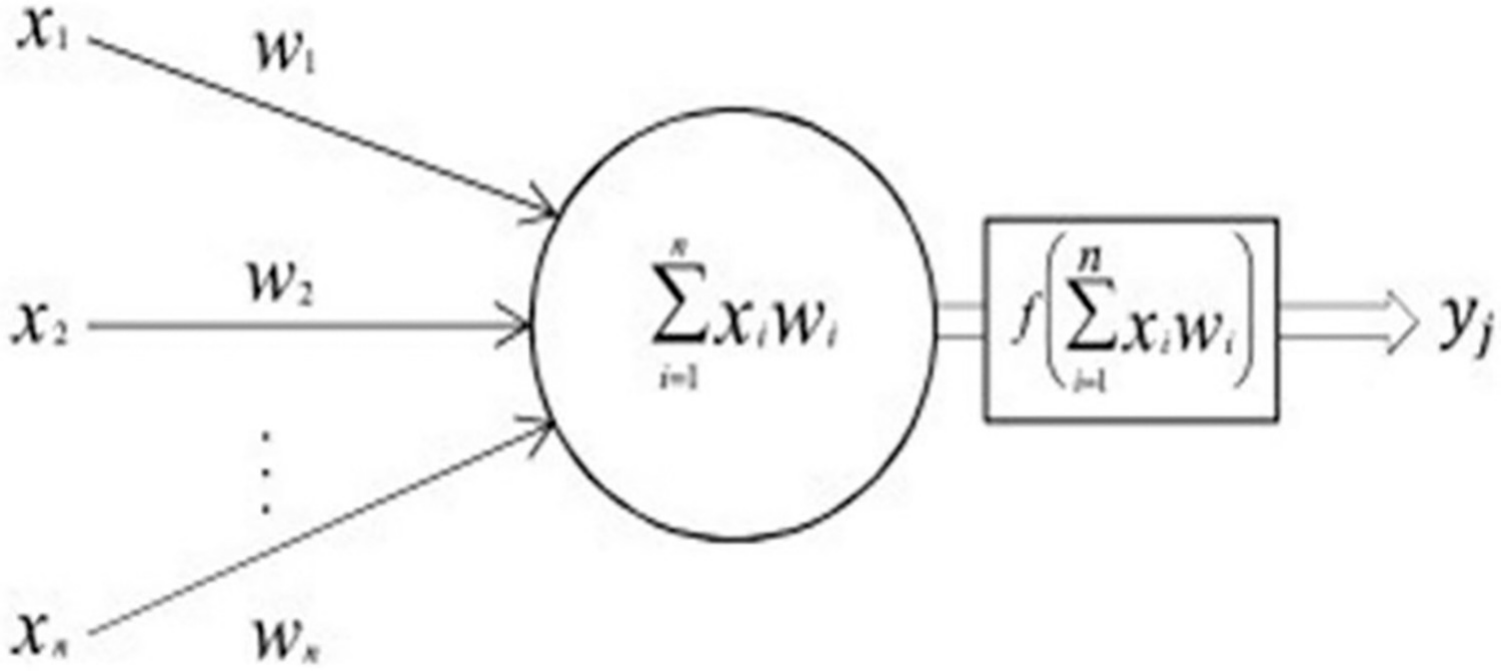
Artificial neuron.

A perceptron is a neural network with one layer (single-neuron network) [20,21] and is normally used for linear binary classification using the sigmoid activation (threshold-based) function. It comprises input, bias, and weight values, as well as an activation function and weighted sum. Fig 5 shows a simple artificial neuron and its components, where the arrows represent connections, *X* represents the input matrix, *W* represents the weight matrix, $\sum_{i=0}^{x}\;x_{i}w_{i}$ is the weighted sum of the input, *f* is the activation function and *y_j_* is the output.

**Fig 6:**
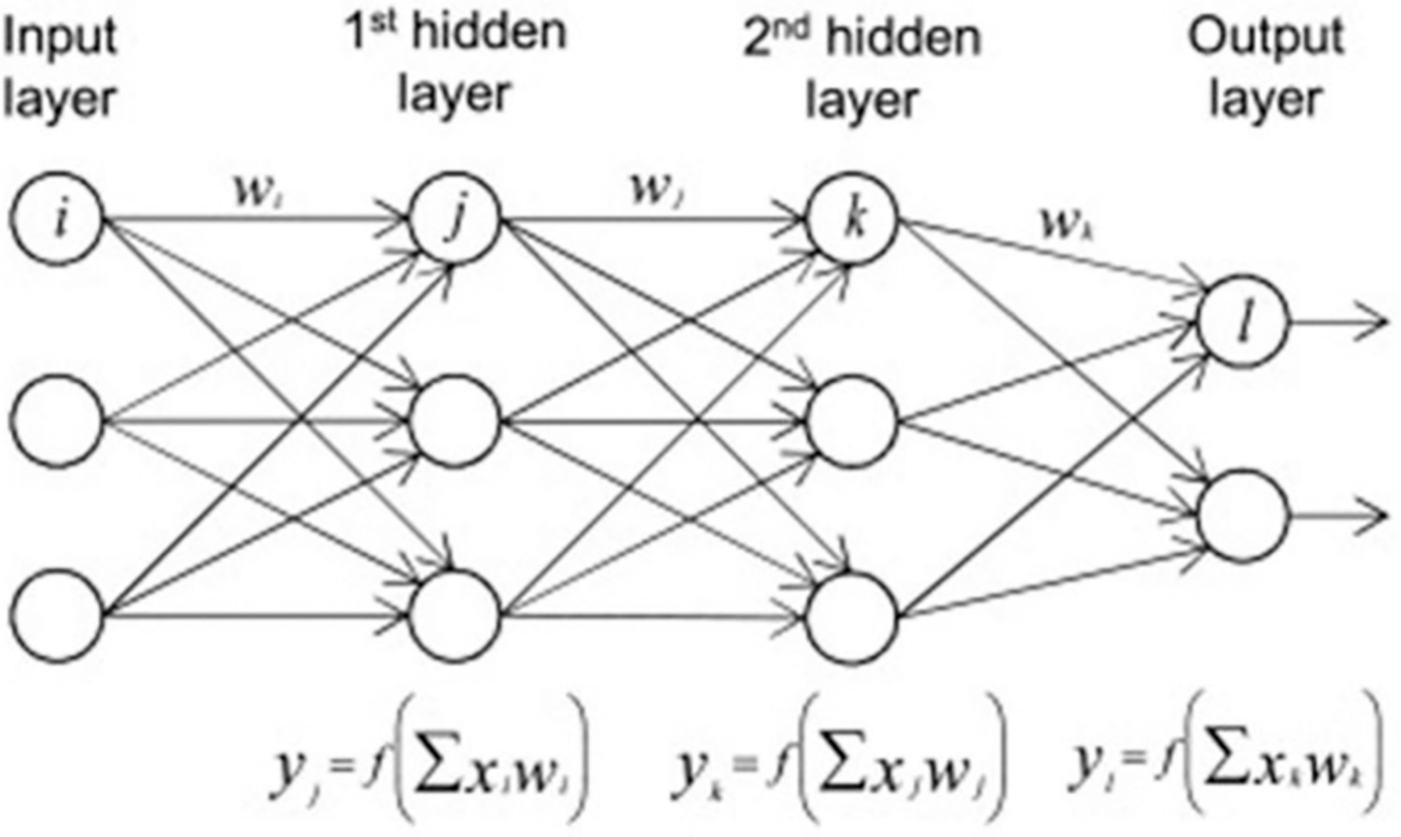
Structure of multilayer perceptron.

Multilayer perceptron (MLP) is a deep learning model that is an extension of the perceptron in that it retains the basic working principle of a perceptron but has multiple layers, unlike a perceptron. Thus, MLP is a fully connected artificial neural network that comprises input, hidden, and output layers, organised in a feed-forward manner [22,23]. The number of hidden layers may vary, and different activation functions may be applied to the hidden neurons, which may be different from the ones at the output layer. For instance, rectified linear units (ReLU) may be applied to the other layers, while the sigmoid is applied to the output layer for binary classification. Fig 6 shows the structure of a multilayer perceptron. However, readers must note the actual number of hidden layers can differ from one model to the other, depending on the problem space. Determining the appropriate number of hidden layers and neurons for a given problem is a part of the hyperparameter tuning (optimization) task. The MLP model was trained using backpropagation and rectified linear units. Fig 6 shows the structure of a multilayer perceptron, which comprises multiple neurons in various layers.

**eXtreme Gradient Boosting** (XGBoost) is an ensemble-based ML algorithm that is based on the Gradient Boosting algorithm and is used to handle linear classifiers and linear regressions [24]. As an ensemble method, it combines multiple weak classifiers to create a powerful and more robust model [2]. It solves classification problems by using a tree-learning algorithm and linear model [25]. Moreover, the power of this algorithm also lies in its ability to be extended to other use cases, such as handling missing values and avoiding overfitting [26]. Thus, the authors used the XGBoost algorithm for STI prediction.

Extremely randomized trees (ExtraTrees) is an ensemble learning model widely used to solve classification and regression tasks. It is an extension of the random forest algorithm that uses bootstrapping, decision trees and voting [27]. To perform bootstrapping, ExtraTrees creates multiple bootstrap samples from sub-samples of the dataset and each sample is generated by randomly selecting data points with replacement, creating subsets of the data [28]. For each bootstrap sample, Extra Trees builds a decision tree, which involves recursively partitioning the data at each node based on feature splits. After constructing all the decision trees, the predictions from each tree are combined to make the final prediction based on majority voting.

### 4.2 Hyperparameter Tuning and performance STI predictive models

The implementation of STI predictive models was done through Python programming and Libraries that support machine learning and deep learning algorithms. The study also applied StratifiedKFold validation to further assess the model’s performance by splitting the data into *k*-subsets (folds). Each STI predictive model was trained and evaluated *k*-times, with each fold serving as the validation set while the others acted as the training data. Hyperparameter tuning was conducted through a randomized grid search (n_splits = 5, shuffle = True), which leverages both the fit and score methods to determine the best parameters. For each STI predictive model, the best parameters were identified and documented as follows:

- MLP’s best parameters: activation: ’relu’, alpha: 0.01, hidden_layer_sizes: (200,), learning_rate: ’adaptive’, solver: ’adam’
- XGBoost’s best parameters: reg_alpha: 0.001, reg_lambda: 10
- ExtraTrees’ best parameters: criterion’: ’entropy’, max_features: ’sqrt’, n_estimators: 150.

**Fig 7:**
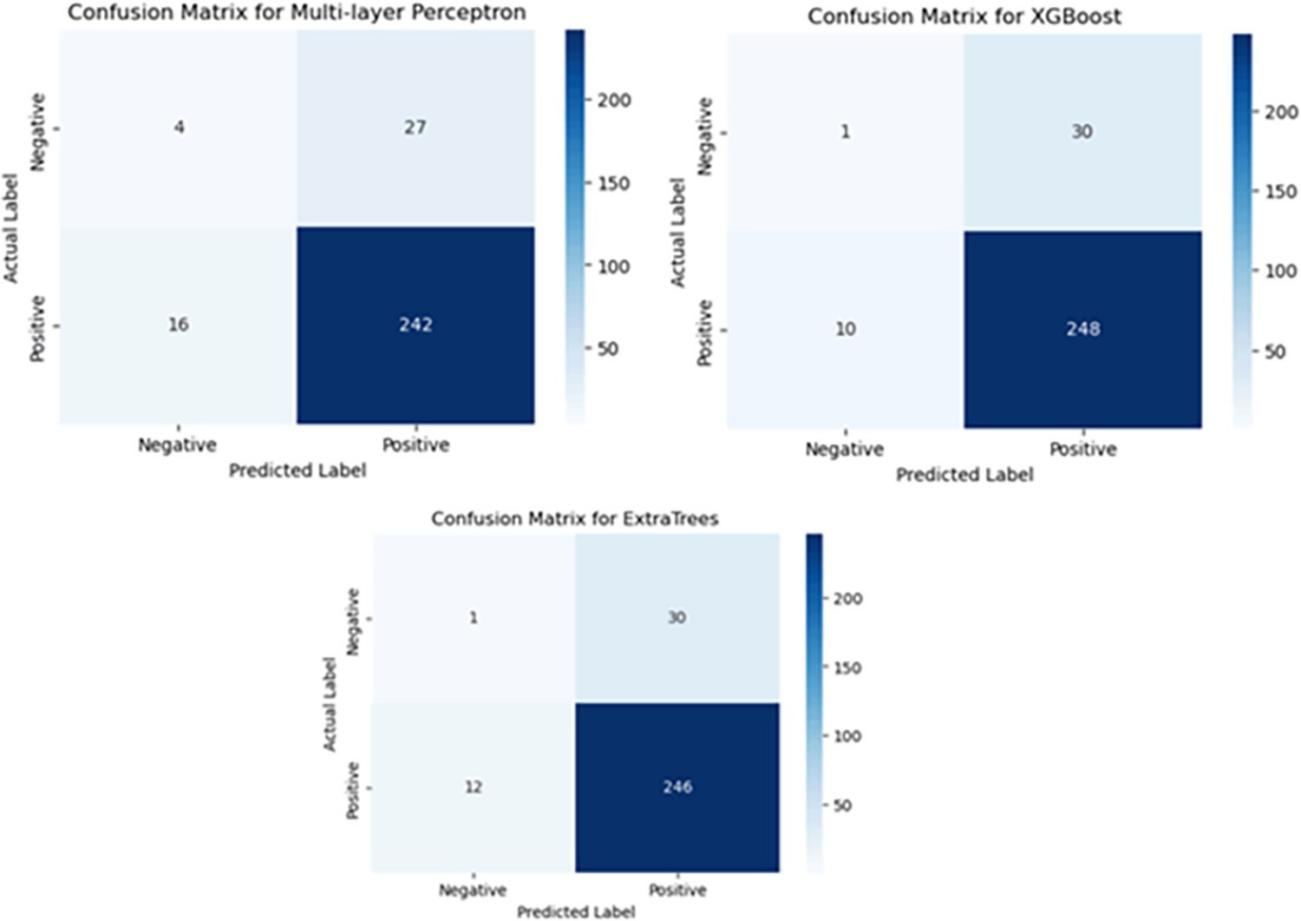
Confusion matrix for STI predictive models.

The performance scores of STI predictive models were assessed based on the confusion matrix (as shown in Fig 7) which computes true positives (TP), true negatives (TN), false positives (FP), and false negatives (FN). From the confusion matrix, we were able to determine the accuracy, recall, F1-score, precision, and AUC of STI predictive models, as shown in Table 3.

**Table 3:** Performance of STI predictive models with SMOTE.

STI predictive model	Accuracy (%)	Recall (Sensitivity) (%)	Precision (%)	F1-Score (%)	Specificity (%)	AUC (%)	P-value
MLP	87.54	97.29	89.64	93.31	6.45	66.78	0.9355
XGBoost	86.51	96.51	89.25	92.74	3.23	54.83	0.9511
ExtraTrees	85.47	95.35	89.13	92.13	3.23	60.21	0.9118

Accuracy measures the overall correctness of the model’s predictions. It is the ratio of correct predictions (TP + TN) to the total number of instances [3]. The study also used recall, which is also known as sensitivity or true positive rate (TPR), which measures the proportion of actual positive instances correctly identified by the model. It is calculated as, (TP / (TP + FN)). We also used the F1-score to determine the performance of STI predictive models. F1-score is the harmonic mean of precision and recall. It provides a balanced measure of the model’s performance, considering both false positives and false negatives [29]. In addition, the study also used precision to determine the proportion of predicted positive STI instances that are positive (TP / (TP + FP)). Specificity or true negative rate (TNR) was also used to evaluate the performance of STI predictive models. Specificity measures the proportion of true negatives out of all actual negative instances [30]. It is calculated as, true negatives / (true negatives + false positives)[31].

The study further used the area under the curve (AUC) to assess the performance of STI predictive models. It represents the area under the Receiver Operating Characteristic (ROC) curve, which plots the true positive rate (TPR) against the false positive rate at various classification thresholds [29]. The TPR is the proportion of true positive STI instances (correctly classified positives) out of all actual positive instances, and the FPR is the proportion of false positive STI instances (incorrectly classified negatives) out of all actual negative instances [27]. The value of AUC is between 0.5 and 1, and closer to 1 indicates the better performance of the model [3]. Therefore, Table 3 shows the performance results of the models with SMOTE.

The results in Table 3 show that MLP performed better than other STI predictive models in terms of accuracy, recall, precision, F1 score and AUC. The MLP model achieved the highest accuracy with a score of 87.54%, followed closely by XGBoost with 86.51%, and ExtraTrees with 85.47%. This indicates that the MLP model had the highest proportion of correct predictions as compared with other models. In terms of recall or sensitivity, MLP model achieved the highest recall score of 97.29%, indicating that the model correctly identified the highest proportion of STI-positive cases. XGBoost followed closely with a recall of 96.51%, while ExtraTrees had a slightly lower recall of 95.35%. Again, MLP achieved the highest precision score with 89.64%, indicating a higher proportion of correctly predicted STI-positive cases out of all positive predictions. XGBoost and ExtraTrees had slightly lower precision scores of 89.25% and 89.13%, respectively.

**Fig 8:**
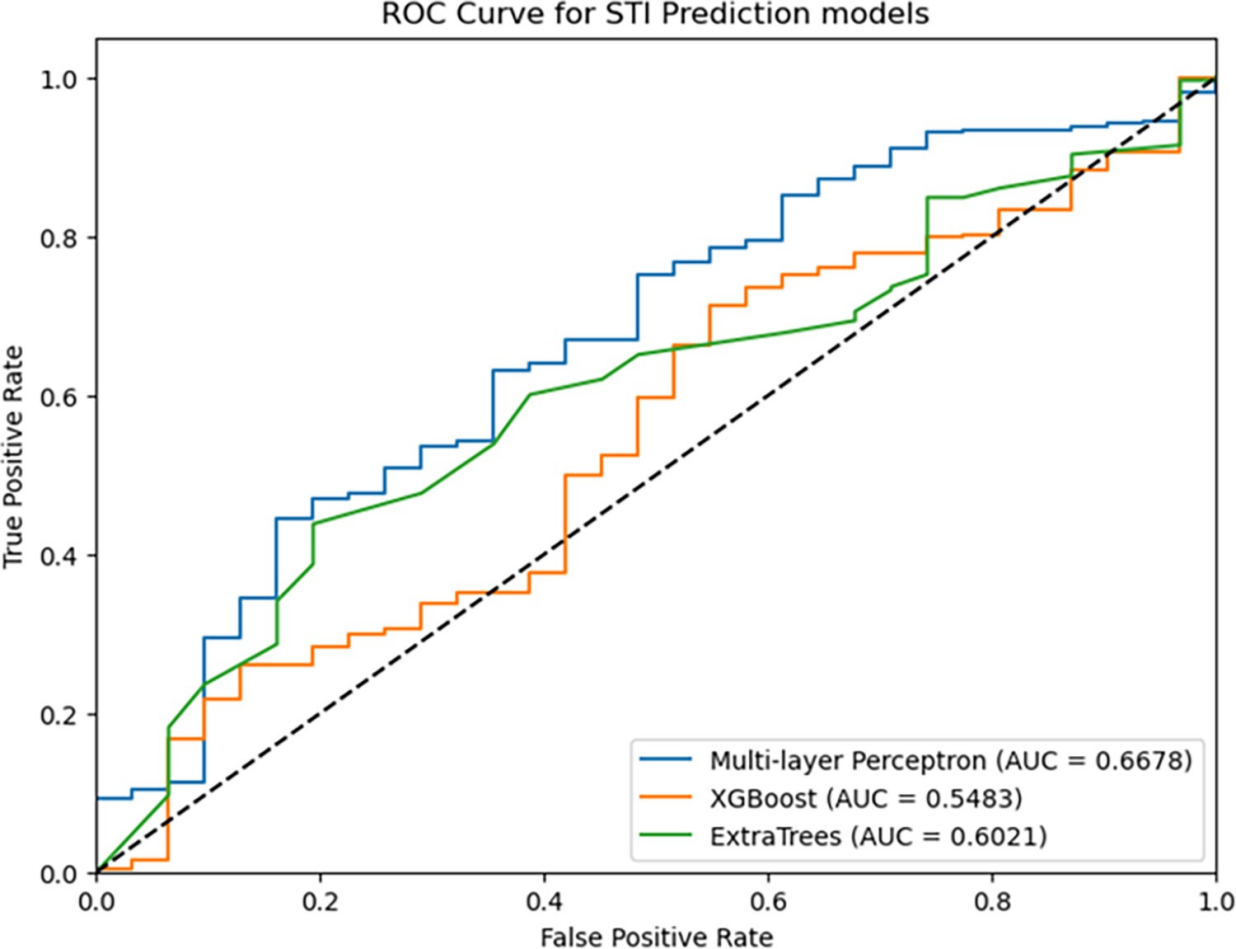
STI predictive models’ AUC values.

Table 3 also shows that MLP achieved the highest F1 score (93.31%), indicating a better balance between precision and recall. XGBoost and ExtraTrees had slightly lower F1 scores of 92.74% and 92.13%, respectively. Additionally, among other models, the MLP model recorded a specificity score of 6.45%. Fig 8 also shows that MLP achieved an AUC value of 66.78%, indicating its performance across various classification thresholds. ExtraTrees recorded AUC of 60.21%, slightly lower than MLP, while XGBoost recorded an AUC of 54.83%. The larger the AUC value, the better the overall performance of the test.

Moreso, the study also considered the p-values associated with the MLP, XGBoost and ExtraTrees models to indicate the statistical significance of their performance. A high p-value indicates that the observed results are unlikely to have occurred by chance. This provides more confidence in the model’s performance, not due to random variations, but rather meaningful and reliable patterns. Therefore, both MLP and XGBoost models have high p-values of 0.9355 and 0.9511, respectively, indicating that their performance is statistically significant. Similarly, the ExtraTrees model also has a relatively high p-value of 0.9118, indicating statistical significance.

## 5. Discussion

The logistic regression analysis conducted in this study aimed to unravel the intricate web of risk factors contributing to sexually transmitted infections (STIs) among Men who have Sex with Men (MSM). The identified variables shed light on key aspects of MSM demographics and behaviours associated with varying levels of vulnerability to STIs. Our findings indicate a heightened susceptibility among individuals aged 26–35 years compared to the reference age group (18–25 years). This aligns with a study conducted in Zhejiang, China, where MSM aged 26–35 years were reported to be 1.95 times more likely to face the risk of syphilis infection compared with those aged 18–25 years [32]. Certain age groups, as highlighted by Beck et al.[33], may represent critical periods where individuals are more prone to engaging in risky sexual behaviours. Pinpointing these age ranges allows for targeted public health interventions to address the unique challenges faced during these periods.

Our study also emphasizes the importance of exploring the timing of first sexual encounters within the MSM community. Those who had their first sexual encounter with a man between the ages of 36–45 years were found to be at a greater risk of STI infection. Cultural and societal factors, as noted by Adedimeji et al. [34], play a role in shaping the timing of sexual initiation. Understanding these influences can inform culturally sensitive interventions and policies tailored to the specific challenges faced by individuals in different communities and social contexts. Early or late involvement in the MSM community may have long-term implications for sexual health, exposing individuals to different risks and health concerns over their lifespan [35]. In addition, our study aligns with research by Kevlishvili et al.[36], which explored the socio-economic features of STIs among MSM. Low-income levels and educational attainment were identified as the main socioeconomic risk factors for high STI prevalence among MSM. Our findings further support this by indicating that individuals with no education or only primary education are more at risk compared to those with secondary, tertiary, and vocational levels of education. Notably, vocational-level education stood out as statistically significant. Supporting these findings, it is recognized that individuals with higher educational attainment may have better access to healthcare resources, including STI testing and prevention services. This access facilitates early detection, timely treatment, and the adoption of preventive measures, thereby reducing overall STI risk. Additionally, education’s influence on how individuals perceive and communicate about sexual risks, as highlighted by Soe et al. [37], underscores the role of education in promoting effective communication about sexual health, contributing to safer practices and reduced STI transmission.

Furthermore, the study applied multilayer perceptron (MLP), ExtraTrees, and XGBoost classifier to predict STIs among MSM using variables (predictors) identified using logistic regression. Predictors such as age, cohabitation with sex partner, education status, employment status, religion, sexual identity, transactional sex, number of sexual partners in the past 6 months, condom use, and HIV status were identified as significant variables. Predictors such as age, condom use, sexual identity, and number of casual male sexual partners were also used by Bao et al. [2] in Australia to predict HIV and STIs among MSM. The identified variables were ranked using the random forest model and revealed that age, age of having first sex with a man, sex partners, religion, and education are among the top most important features with high-importance feature scores. The study reveals that MLP performed better in predicting STIs among MSM with a high accuracy of 87.54%, recall of 97.29%, precision of 89.64%, F1-Score of 93.31% and AUC of 66.78%. Other studies, including one by Birri Makota and Musengi [14] also applied different HIV prediction models and XGBoost outperformed other models. With such good performance, these models can be effectively used to develop pre-test STI infection screening tools to identify highly at-risk individuals within the MSM community [2] and prioritise resource allocation to improve the health outcomes of these hard-to-reach communities. Improving HIV and STI diagnosis among MSM is paramount to achieving the World Health Organization (WHO) target of reducing new HIV infections to less than 560,000 by 2025 [38]. This is also supported by a study conducted by Bao et al.[2] which alluded to the need for integrating HIV and STIs (syphilis, gonorrhoea, chlamydia) predictive models into health systems. Based on the findings of this study and existing literature, there is a need to integrate intelligent machine learning models such as XGBoost into the existing health information systems to assist healthcare professionals with early screening of STIs especially among MSM. Furthermore, such advanced intelligent models can be utilized to develop data-driven tools for key population groups including MSM to enhance STI screening, reporting and communication, especially in resourced-constrained areas and in countries where same-sex sexual behaviours are highly stigmatised and criminalised. Integrating STI predictive models with existing health information systems can assist health workers in providing care to MSM. Even though STI predictive models show promising results, there is a need to further improve their performance and validate and implement them in the real world as a pre-test STI screening tool to improve the health outcomes of MSM.

## 6. Limitations of the study

This study had several limitations. One of the limitations is that the sample size and geographic focus of the study data was limited to the urban provinces of Bulawayo and Harare and is therefore not representative of the other rural provinces of the country. Therefore, the survey does not reflect MSM and TGW/GQ activity throughout all of Zimbabwe. Another limitation is that the study was cross-sectional and therefore, participants were not followed up over time. By their nature, cross-sectional studies do not show a temporal relationship between exposures and outcomes as longitudinal studies would. The third limitation is that all questionnaire data were self-reported and may have been subject to social desirability bias. Although interviewers were trained in techniques to put participants at ease and support the accurate reporting of dates and events, self-reported data remain susceptible to recall bias and social desirability bias. The fourth limitation is that same-sex sexual behaviours are illegal and highly stigmatised in Zimbabwe. Those who were recruited and agreed to participate may be a self-selected group of individuals more comfortable disclosing their sexual behaviour.

## 7. Policy Recommendations

A new report from UNAIDS shows that Zimbabwe is one of the five sub-African countries having already achieved the targets 95-95-95 and is on track to end AIDS by 2030. However, there are key indications that Key populations in particular MSM are lagging in the attainment of the 95-95-95 by a large margin [39]. Approaches such as those described in this article will go a long way in assisting the Zimbabwe Ministry of Health and Child Care in predicting sexually transmitted infections among MSM in the country and providing the necessary health education and STI screening tests to the individuals at the highest risk of STI acquisition. This strategy will assist in ensuring that these individuals will be less likely to acquire HIV and STI and ensure that those who are positive for these infections are timeously provided with treatment and care services. The approaches outlined in this article have relevant applications in other southern African countries where sizable MSM populations exist and where activities of this group are illegal just like in Zimbabwe and where MSM do not visit health facilities as frequently as they should out of fear of stigma and harassment by law enforcement authorities. It is key that on the limited occasions MSM visit a health facility they are provided with all the medical attention they require as well as screened for infectious conditions such as STIs [39,40]. The approaches in this article should be in health ministry policies and guidelines to ensure that the model is fully utilised.

## 8. Conclusion

Integrating machine learning and deep learning can play an important role in reducing new HIV/STI infections among men who have sex with men. This study shows that age, cohabitation with a sex partner, education status, employment status, religion, sexual identity, transactional sex, number of sexual partners in the past 6 months, condom use, and HIV status are among important predictors for predicting STIs among MSM in Zimbabwe. The study further revealed that MLP achieved accuracy of 87.54%, recall of 97.29%, precision of 89.64%, F1-Score of 93.31% and AUC of 66.78%. MLP model generally outperformed the XGBoost and ExtraTrees models across most metrics, showing higher accuracy, recall, precision, F1-score and AUC. These models can be effectively used to identify highly at-risk individuals within the MSM community and further develop STI infection screening tools to improve surveillance health outcomes of MSM, especially in countries where it is stigmatised and criminalised.

In conclusion, our findings provide nuanced insights into the multifaceted landscape of STI risk among MSM in Zimbabwe. By shedding light on age dynamics, the timing of first sexual encounters, sexual partner dynamics, and educational attainment, this study contributes to a more comprehensive understanding of the factors influencing STI prevalence in the MSM community. The implications extend beyond statistical associations, emphasising the need for comprehensive and tailored public health strategies. As with any observational study, further research and validation are essential to corroborate these findings and refine our understanding of the complex dynamics influencing STI prevalence among MSM.
